# The dynamic characteristics of the shearer cable drag system

**DOI:** 10.1371/journal.pone.0316319

**Published:** 2024-12-31

**Authors:** Bo Xie, Feida Wang, Jing Liu, Zhanjun Xu, Shuaishuai Gao, Lijuan Zhao

**Affiliations:** 1 Shandong Yankuang Group Changlong Cable Manufacturing Co., Ltd, Jining, China; 2 School of Mechanical Engineering, Liaoning Technical University, Fuxin, China; 3 The State Key Lab of Mining Machinery Engineering of Coal Industry, Liaoning Technical University, Fuxin, China; 4 Liaoning Province Large Scale Industrial and Mining Equipment Key Laboratory, Fuxin, China; King Fahd University of Petroleum & Minerals, SAUDI ARABIA

## Abstract

Based on the 5615 working face of Beisu Coal Mine, a virtual prototype of the shearer cable drag system was developed using the MG2×70/325-BWD electric traction shearer as the carrier, in combination with CERO and ADAMS software. The shearer cable was equivalently modeled using the discrete rigid body method to study the dynamic characteristics of the drag system. This research provides a foundation for the design and optimization of both the cable and cable clamps. The results indicate that during the bending process of ordinary and reinforced cables in the cable drag system, the tensile force between the cable clamps increases from approximately 28 N and 37 N to a maximum value of 133 N and 146 N, respectively, before decreasing to around 57 N and 66 N. At the connection point between the drag system and the shearer, the tensile force between the cable clamps fluctuates and increases, reaching a maximum value of 925.2 N and 1134.7 N when the shearer reaches the end of the working face. These values are significantly lower than the cable clamp’s breaking tensile strength of 70 kN, with peak values of 57.4 N and 94.1 N, respectively. During the cable bending process, The contact force with the cable clamp continuously changes with the bending angle, During 0 ~90°, the contact force between ordinary and reinforced cable and cable clamp gradually increases to the maximum values 61.3N and 86.2N, After 90 have plummeted to near 23 N and 25 N, In the process of dragging the cable to the top of the roller, it fluctuates between 45.2~51.7 N and 66.3~73.6 N respectively, Cable exit bends are slowly reduced to fluctuations around 16 N and 17 N, The tensile force between ordinary and reinforced discrete cables increased to around 58.4 N and 80.5 N and then decreased to around 12.6 N and 32.8 N, respectively. During the bending process of reinforced cables, the average contact force with the cable clamps increased by 22.3 N compared to the ordinary cables, while the average discrete tensile force between the cables increased by 18.7 N. Although the tensile and contact forces of the reinforced cable are slightly higher than those of the ordinary cable, the reinforced cable has a higher safety factor and greater adaptability. The drag motion of the cable is correlated with the characteristics of the ring-chain drive, and the drag speed and tensile force exhibit periodic variations due to the polygonal effect of the chain drive. The findings provide valuable insights for intelligent cable drag research and lay the foundation for the optimization of mining cables and cable clamps.

## 1. Introduction

With the development of intelligent coal mining, both efficiency and safety in coal extraction have significantly improved. As the core carrier for power supply to coal mining machines, cables are critical for ensuring reliable power transmission and stable operation of the shearer [1, 2]. In thin coal seam working faces, the operating environment for shearer cables is harsh. The cable system experiences multiple layers of overlap during the reciprocating motion of the shearer, affecting both cable longevity and the safety of the cable dragging process. Intelligent cable drag systems are increasingly being applied in fully mechanized working faces. These systems transmit power to the cable drag cart through a drive mechanism, achieving the goal of cable traction. This not only enhances safety but also reduces labor requirements and increases efficiency, further advancing the intelligence of fully mechanized equipment [3].

Domestic and international scholars have conducted research on the issue of cable dragging: Shi Gang et al.[4] proposed an identification method for the extrusion pressure of shearer dragging cables based on an improved deep forest algorithm, addressing the challenge of assessing the stress endured by ethylene-propylene rubber insulated cables. The Black Dragon system developed by the Polish Kopex Group [5] was equipped for the first time with a coal miner cable towing device, in which the towing of the coal miner cables is controlled by a guide wheel, avoiding the need for multiple layers of cables to be stacked. Li Yuhua et al. [6] designed an overload protection device for shearer cable traction to address issues such as excessive pulling force, cable damage, and traction of cable clamps. Hao Yuanyuan [7] designed a shearer cable dragging system that analyzes the stress distribution patterns of chain links under different loads. The Zhangjiakou Coal Mine of China Coal showcased an intelligent linkage device for shearer cables at the 19th China International Coal Mining Technology Exchange and Equipment Exhibition, which implements cable dragging through a laterally arranged chain transmission system [8].

In the cable dragging system, the cable acts as a highly deformable flexible component with a multi-level twisted structure, making direct simulation challenging. Therefore, appropriate methods are required for simplification. You Bindi et al. [9] established a dynamic model for highly flexible cables based on the theory of elastic slender rods, analyzing the mechanical properties of these cables under large deformations. Wang Xiaoyu et al. [10] modeled and simulated the cables using the absolute nodal coordinate method, assessing the impact of various factors on the motion of highly deformable cable bodies. Tang Lu [11] employed a method using sleeve force connections for discrete rigid bodies to study the simulation of highly flexible steel cables, validating the feasibility of this approach. Wang Yingjun [12] derived the formula for the equivalent elastic modulus of twisted structures and introduced the concept of the shape coefficient for steel wire ropes. R. N. A. Algburi et al. [13, 14] proposed a novel method combining singular spectrum analysis with a hierarchical hyper-Laplacian prior model. Using data collected from rotary encoder sensors, this approach can evaluate the health and performance of robotic systems, monitor and diagnose cable faults in industrial robots, and extract weak fault features from vibration signals with significant noise interference. By integrating singular spectrum analysis and a generalized structured shrinkage algorithm, the method effectively identifies noise interference, discrete frequency disturbances, and cyclic pulses. This enhances fault feature recognition, optimizes diagnostic outcomes, and ensures the reliability of cable connections.

Current research on cable dragging systems primarily focuses on model design and the analysis of individual components [15–17]. However, the impact of cables on system operation has not been considered, and the interactions among various components have been overlooked. Therefore, further exploration of the overall dynamic characteristics of the system is needed. This study incorporates a discrete cable model into the drag system, addressing the challenge of capturing the dynamic characteristics of the cables. Virtual prototyping techniques are employed to investigate the overall motion of the drag system, exploring the interactions between the cable clamps and cables, as well as the impact of the chain drive on the drag system. The findings help designers understand the operational state of the cables under real-world conditions, provide dynamic characteristic data under working conditions, and lay the foundation for the design and optimization of shearer power cables and cable clamps.

## 2. Construction of an equivalent cable model

### 2.1 Tensile test of cable strands

Taking the MCP-0.66/1.14 (395+135+4*10) cable as the engineering subject, tensile tests were conducted on the power core, ground core, and control core using a tensile testing machine. The mechanical parameters of each core were obtained through analysis of the test results.

**Fig 1:**
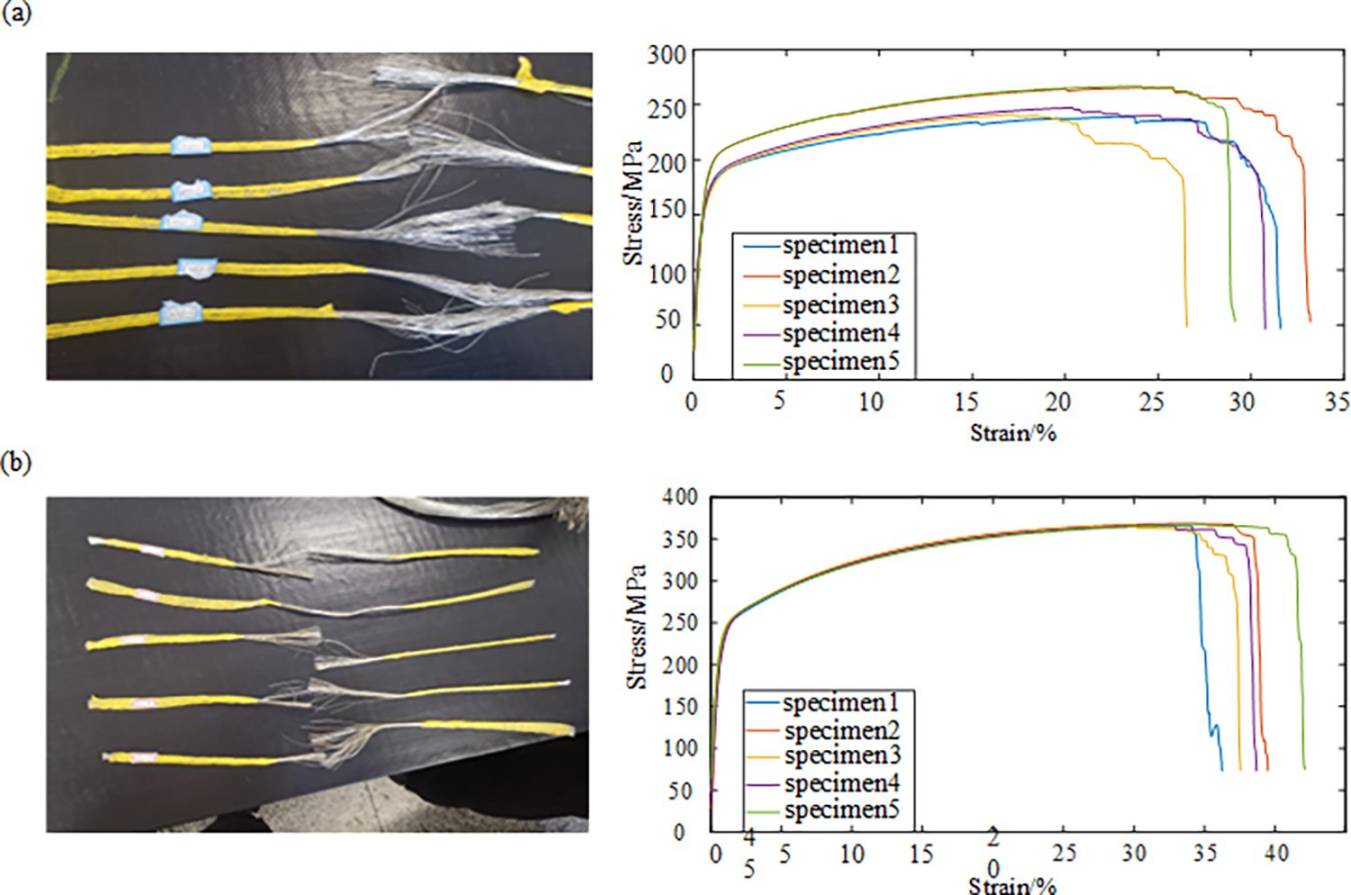
Tensile test samples of power core, ground core, control core: (a) Power and ground cores; (b) Control core.

Five sets of samples were taken from the power core, ground core strands, and control core, with a clamping length of 30 mm and a tensile rate of 0.1 m/min. Tensile data were collected every 0.02 s. The test was carried out until the sample broke and the stress-strain curve was plotted as shown in Fig 1.

The strand and control wire core specimens have no obvious yielding phenomenon during the tensile process, and their yield strength can be expressed by the non-proportional elongation strength. The data of elastic and plastic deformation stages in the stress-strain curve of the tensile test were fitted to obtain their elastic and tangential moduli respectively, and the experimental data of each group were processed and averaged, and the results of data processing are shown in Table 1.

**Table 1:** Femoral tensile test data.

Strand type	Modulus of elasticity *E*_0_/GPa	Yield strength /MPa	Tangent modulus *E*_T_/GPa	Tensile strength/MPa
Power and ground core strands	30.2	241.4	801.7	354.4
Control core	24.4	182.7	567.7	247.1

### 2.2 Equivalent cable parameter calculation

**Fig 2:**
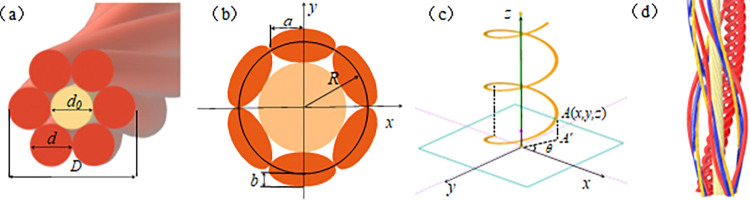
Schematic diagram of cable structure: (a)Theoretical diameter of the Cable Core; (b) Stranding size of the cable Core (c) Helical stranding of the wire Strand (d) Helical stranding of the cable core.

The cores consist of strands bundled together, with tightly twisted copper wires. The bundle of strands serves as the smallest unit of the cable. The first-level stranding structure involves six outer bundles of strands twisted around a central bundle, as shown in Fig 2(A). The actual dimensions after stranding are shown in Fig 2(B).

For ease of analysis, a Cartesian coordinate system, as shown in Fig 2(C), was established to analyze the equivalent elastic modulus of the core. The internal structure of the conductor was built layer by layer, as shown in Fig 2(D), to study its equivalent elastic modulus.

Helix for the centre line of the outer wire, axial z direction, its cross-section for the xoy plane. Cable strands stranded, to pitch as a unit of periodic change, take the strands of any point A (x,y,z), the point in the xoy plane projection point for A′, where the coordinates of the A′ point as shown in Eq (1).

$$\{\begin{array}{l} x=R\cos\theta \\ y=R\sin\theta \\ z=\theta /2\pi L \end{array}$$ (1)

where *R* is the radius of the strand; *d* and *d*_*0*_ are the radius of the centre strand and the first-stage strand, respectively; *θ* is the corresponding polar angle at point *A′*; and *L* is the strand pitch.

Taking the micro-arc *A′B* over the point *A′*, the coordinates of the point *B* are (*x + dx*,*y + dy*,*z + dz*), so we have:

$$\left\{ \begin{array}{l} dx=-R\sin\theta d\theta \\ dy=R\cos\theta d\theta \\ dz=L/2\pi d\theta \end{array}\right.$$ (2)

From Eq (2), the length of the microarc *MN* is *ds* and the strand length *S* is shown in Eq (3).


$$S=\int_{0}^{2\pi }ds=\sqrt{\pi^{2}{\left(d+d_{0}\right)}^{2}+L^{2}}$$ (3)

The cosine of the twist angle of the strand and the nominal area of the strand are shown in Eq (4).


$$\left\{ \begin{array}{l} \cos\alpha =\frac{dz}{ds}=\frac{L}{\sqrt{4\pi^{2}R^{2}+L^{2}}}=\frac{L}{S}\\ A_{0}=6\frac{A_{1}}{\cos\alpha }+A_{2}=\frac{\pi (6Sd^{2}+Ld_{0}^{2})}{4L} \end{array}\right.$$ (4)

Where *A*_*0*_ is the nominal area of the stranded wire, mm^2^; *A*_*1*_ is the outer strand area, mm^2^; *A*_*2*_ is the centre strand area, mm^2^.

To calculate the equivalent modulus of elasticity of the stranded core, the following assumptions are made: the end faces of the strands are perpendicular to the tangent line corresponding to the centre strand; the friction between the wires is neglected; and the strands are taken in torsionless stretching, with the elongation recorded as *ΔL*.

The change in length of the helical wire is calculated as shown in equation (5) [12].


$$\left\{ \begin{array}{l} 1+\frac{4\pi^{2}R}{L}\frac{\Delta R}{\Delta L}\approx 1\\ \Delta S=\frac{\partial S}{\partial R}\Delta R+\frac{\partial S}{\partial L}\Delta L=\frac{L}{S}\Delta L \end{array}\right.$$ (5)

The resultant force of the lateral strand along the Z-axis is expressed in Eq (6).

$$T_{1}=E_{0}\frac{\Delta S}{S}A_{1}\cos\alpha =\frac{\pi E_{0}d^{2}L}{4S^{2}}\Delta L$$ (6)

where *T*_*1*_ is the combined force of the side strands along the *Z*-axis, N; *E*_*0*_ the modulus of elasticity of the strands, MPa. the central strand pulls as shown in Eq (7) after an elongation *ΔL*.

$$T_{2}=\frac{E_{0}A_{2}}{L}\Delta L=\frac{\pi E_{0}d_{0}^{2}}{4L}\Delta L$$ (7)

where *T*_*2*_ is the combined force of the centre strands along the *Z*-axis, N. The tension *T* of the stranded wires is obtained by combining the tensile forces on the six peripheral strands and one centre strand as shown in Eq (8).


$$T=6T_{1}+T_{2}=\frac{TE_{0}}{4}(\frac{6d^{2}L}{S^{2}}+\frac{d_{0}^{2}}{L})\Delta L$$ (8)

The equivalent elastic modulus of the strand is derived from Eq (9), which also allows for the determination of the shape factor *β*。

$$E_{1}=\frac{T}{A_{0}\varepsilon }=\frac{TL}{A_{0}\Delta L}=E_{0}\frac{6d^{2}L^{3}+d_{0}^{2}S^{2}L}{6d^{2}S^{3}+d_{0}^{2}S^{2}L}$$ (9)

Where *E*_*1*_ is the equivalent modulus of elasticity of the stranded wire, MPa. The shape factor and equivalent modulus of elasticity of each layer of the powerline core are calculated step by step.

The elongation and tension of the formed cable is obtained by the combined calculation of the power core, ground core and control core, as shown in Eq (10).


$$T=(\frac{\pi E_{1}}{4}\frac{3{d_{1}}^{2}L}{S^{2}}+\frac{\pi E_{2}}{4}\frac{{d_{2}}^{2}L}{S^{2}}+\frac{\pi E_{0}}{4}\frac{d_{0}^{2}}{L})\Delta L$$ (10)

The equivalent elastic modulus of the cable core is obtained through recursion, as shown in Eq (11).


$$E_{2}=\frac{3E_{1}{d_{1}}^{2}L^{3}+E_{2}{d_{2}}^{2}L^{3}+E_{0}d_{0}^{2}S^{2}L}{3{d_{1}}^{2}S^{3}+{d_{2}}^{2}S^{3}+d_{0}^{2}S^{2}L}$$ 11

Where *E*_*2*_ is the equivalent modulus of elasticity of the wire core, MPa.

The overall material properties of the cable can be regarded as orthogonal anisotropy, the conductor undergoes a single state of tensile deformation, the cable equivalent modulus of elasticity according to the core and the outer layer of insulation elongation of the same assumptions derived from the conditions, as shown in Eq (12).


$$E=\frac{A_{s}E_{s}+A_{a}E_{a}}{A_{s}+A_{a}}$$ (12)

**Fig 3:**
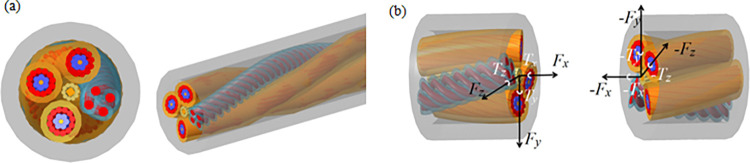
Discrete cable sleeve force modelling:(a) Cable 3 D model; (b) Force model between two cells.

Through the trajpar function, the spiral curve of the wire core is determined according to Eq (1) [18], and the equation of the stranding parameter of the wire core is constructed as shown in Eq (13), and the constructed cable model is shown in Fig 3(A).


A=R+rsin(t×360×N) (13)

In the equation, *R* represents the bending radius of the cable in millimeters (mm); *r* denotes the stranding radius of the helical line in millimeters (mm); and *N* is the number of stranding cycles.

The structure of the cable model is complex; thus, establishing a 1:1 model based on the stranding structure for import into ADAMS would result in a significant computational load. Therefore, a discrete cable approach is adopted, with the segments of the discrete cable connected using shaft forces. In the drag cable system, the installation center distance of the cable clamp is 125 mm. To refine the cable model for easier assembly with the cable clamp, the center distance of the cable clamp must be an integer multiple of the discrete cable length, which is set to 25 mm, resulting in five discrete cable segments forming one group. The material properties of the cable model are defined, yielding an average density of 2.63×10^3^ kg/m^3^ and a mass of 0.79 kg for each discrete segment. To accurately simulate the actual motion state of the cable, the flexible connection is established by defining force components that describe the interaction force and relative deformation between the two discrete cables. The three force components {*Fx*, *Fy*, *Fz*} and the three torque components {*Tx*, *Ty*, *Tz*} between the two units are illustrated in Fig 3(B), and their relationship is expressed in Eq (14) [19].


$$\left[\begin{array}{l} F_{x}\\ F_{y}\\ F_{z}\\ T_{x}\\ T_{y}\\ T_{z} \end{array}]=[\begin{array}{cccccc} K_{11} & 0 & 0 & 0 & 0 & 0\\ 0 & K_{22} & 0 & 0 & 0 & 0\\ 0 & 0 & K_{33} & 0 & 0 & 0\\ 0 & 0 & 0 & K_{44} & 0 & 0\\ 0 & 0 & 0 & 0 & K_{35} & 0\\ 0 & 0 & 0 & 0 & 0 & K_{66} \end{array}][\begin{array}{l} l_{x}\\ l_{y}\\ l_{z}\\ \theta_{x}\\ \theta_{z}\\ \theta_{z} \end{array}]-[\begin{array}{cccccc} C_{11} & 0 & 0 & 0 & 0 & 0\\ 0 & C_{22} & 0 & 0 & 0 & 0\\ 0 & 0 & C_{33} & 0 & 0 & 0\\ 0 & 0 & 0 & C_{44} & 0 & 0\\ 0 & 0 & 0 & 0 & C_{55} & 0\\ 0 & 0 & 0 & 0 & 0 & C_{66} \end{array}][\begin{array}{l} V_{x}\\ V_{y}\\ V_{z}\\ \omega_{x}\\ \omega_{y}\\ \omega_{z} \end{array}]+[\begin{array}{l} F_{1}\\ F_{2}\\ F_{3}\\ T_{1}\\ T_{2}\\ T_{3} \end{array}\right]$$ (14)

Where *l*, *θ*, *v*, *ω* denote the relative displacement, angle of rotation, velocity, angular velocity between the two cables, subscripts *x*, *y*, *z* denote the *X*, *Y*, *Z* coordinate directions, *F*_*1*_, *F*_*2*_, *F*_*3*_ and *T*_*1*_, *T*_*2*_, *T*_*3*_ denote the initial values of the three directional forces and moments, respectively, and *K*, *C* denote the stiffness and damping coefficients, respectively. The stiffness coefficients are derived from the cable equivalent modulus of elasticity as shown in Eq (15).


$$\left\{ \begin{array}{l} K_{11}=\frac{EA}{L}\\ K_{22}=K_{33}=\frac{GA}{L} \end{array}\;\{\begin{array}{l} K_{44}=\frac{\pi GD^{4}}{32L}\\ K_{55}=K_{66}=\frac{EI}{L} \end{array}\right.$$ (15)

Where *K*_*11*_ is the tensile rigidity factor; *K*_*22*_, *K*_*33*_ is the shear rigidity factor; *K*_*44*_ is the torsional rigidity factor; *K*_*55*_, *K*_*66*_ is the bending rigidity factor; *E* is the cable modulus of elasticity, MPa; *G* is the cable shear modulus, MPa; *A* is the cable cross-sectional area, mm^2^; *D* is the cable diameter, mm^2^; *I* is the moment of inertia of each section of the cable, m^4^; *L* is the length of each section of the cable, mm^2^; *C* is the cable damping coefficient.

The cable material is considered to be orthogonal anisotropic, and the results of equivalent modulus of elasticity calculations are combined to obtain the three directional stiffnesses and torques of the quill force.

## 3. Virtual prototyping and modeling of the shearer cable dragging system

Using the MG2×70/325-BWD electric traction shearer as the platform, a 3D solid model of the shearer cable drag system was developed in Creo, based on the performance parameters of mining cables and the spatial structure of the thin coal seam working face. The shearer leads the process, while the cable drag cart follows to handle the cable. The main technical specifications of the shearer are shown in Table 2.

**Table 2:** Technical parameters of the shearer.

Traction method	Maximum traction force/KN	Traction speed/m/min	Supply voltage /KV
Electric traction	2×140	0~6.58~10.96	1.14

The designated traction speed of the shearer is 6.6 m/min. The cable drag system uses chain-driven transmission to operate the cable drag cart, with the cart moving at half the speed of the shearer, i.e., 3.3 m/min. Based on this, the angular velocity of the sprocket is calculated to be 4.42 r/min. The specifications of the chain drive components are listed in Table 3.

**Table 3:** Chain drive specification parameters.

Circular chain specifications /mm	Number of sprocket teeth/*N*	Chain wheel pitch is round and straight *D*_0_/mm	Outer diameter of sprocket *D*_e_/mm
18×64	6	248	284

The system uses O-type cable clamps, with the casing made of nylon 66 and the internal framework made of No. 20 steel [20].

The shearer cable towing system includes three sub-systems: cable clamp towing system, power supply cable system, and circular chain drive system, and the contact and constraints are added in batch using macro commands in ADAMS [21], and the sprocket speed of the start-up state is set as shown in Eq (16).


step(x,a,b,c,d) (16)

Where *x* is the function variable, *a* is the initial time, *b* is the initial value of the sprocket rotational speed, *c* is the ending time and *d* is the final value of the sprocket rotational speed. The sprocket rotational speed is incremented from 0 to 4.42 r/min from 0 to 0.5s, and remains constant from 0.5 to 5s.

The circular chain system serves as the power component of the dragging system, with defined contacts between the chain links and between the chain links and the sprockets. The cable clamps are connected through axial force, with specific parameters provided in Table 4.

**Table 4:** Simulation-related parameters.

Name	Contact Stiffness /N/m	Force Index	Damping /N·s/m	Coefficient of Kinetic Friction	Tensile Stiffness /N/m	Tensile Damping /N·s/m	Torsional Stiffness /N/m	Torsional Damping /N·s/m
Parameters	2.0×10^7^	1.5	500	0.1	7.1×10^5^	115	2.9×10^7^	171

**Fig 4:**
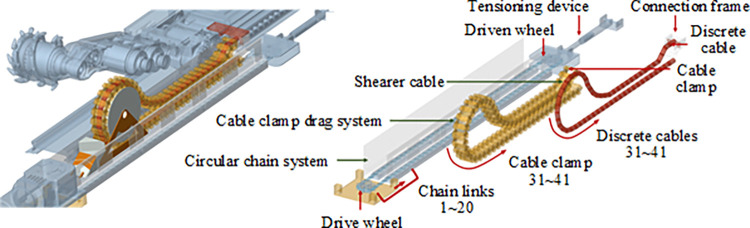
Virtual prototype of the shearer cable towing system: (a) Virtual Prototype of the Cable Dragging System (b) Composition and Numbering of the Cable Dragging System.

Constraints between the cable clamps are added through a double-loop command stream. The shearer is simplified by incorporating constraints for the drive components and assembling it with the dragging system. The constructed virtual prototype of the shearer cable dragging system is illustrated in Fig 4(A). To facilitate macro command compilation and subsequent research, the discrete cables, cable clamps, and circular chains are numbered as shown in Fig 4(B). The vibrations of the cables and cable clamps at the connection point between the shearer and the dragging system are the most intense, while the stress conditions at the cable bends are the most complex; therefore, segments 1–3 and 31–41 are selected as the primary research subjects.

**Fig 5:**
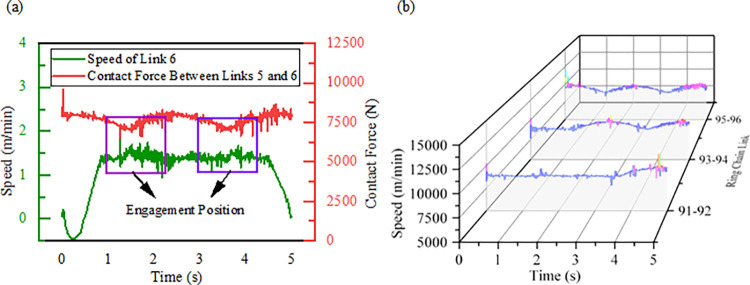
Link engagement speed and contact force curves:(a) Link engagement speed; (b) Link engagement contact force.

Based on virtual prototyping technology and cable design parameters, the model is appropriately simplified while respecting the cable prototype and tensile test data [22]. The drag system is powered by a ring chain, and a virtual prototype model is constructed through theoretical calculations [23], supplemented by relevant literature. Simulations are conducted according to the given operating conditions [24], with the simulation results shown in Fig 5.

The engagement process is influenced by the polygonal effect of the chain drive, and the periodic variation in the contact force reflects the link engagement process. The magnitude of the contact force changes periodically with the engagement and disengagement of the links, which is consistent with the actual transmission behavior of the ring chain [25]. The comparison of the mean values of the meshing contact force and the mean values of the meshing speed is shown in Table 5, and the error of the simulation results is less than 5%. The model is able to meet the simulation accuracy requirements and simulate the performance of the towing cable system under operating conditions [26].

**Table 5:** Comparison of simulation results.

Simulation model	Mean value of engagement contact force	Mean value of engagement speed
Findings of this paper	7534N	1.439
Literature findingsSystem	7340N	1.415
Error/%	2.643	1.696

## 4. Simulation analysis of the dynamic characteristics of the cable dragging system

Based on the constructed virtual prototype model of the cable dragging system, analyze the motion laws and force conditions of each subsystem within the cable dragging system to obtain the system’s dynamic characteristics.

### 4.1 Dynamic characteristics analysis of the cable clamp dragging system

**Fig 6:**
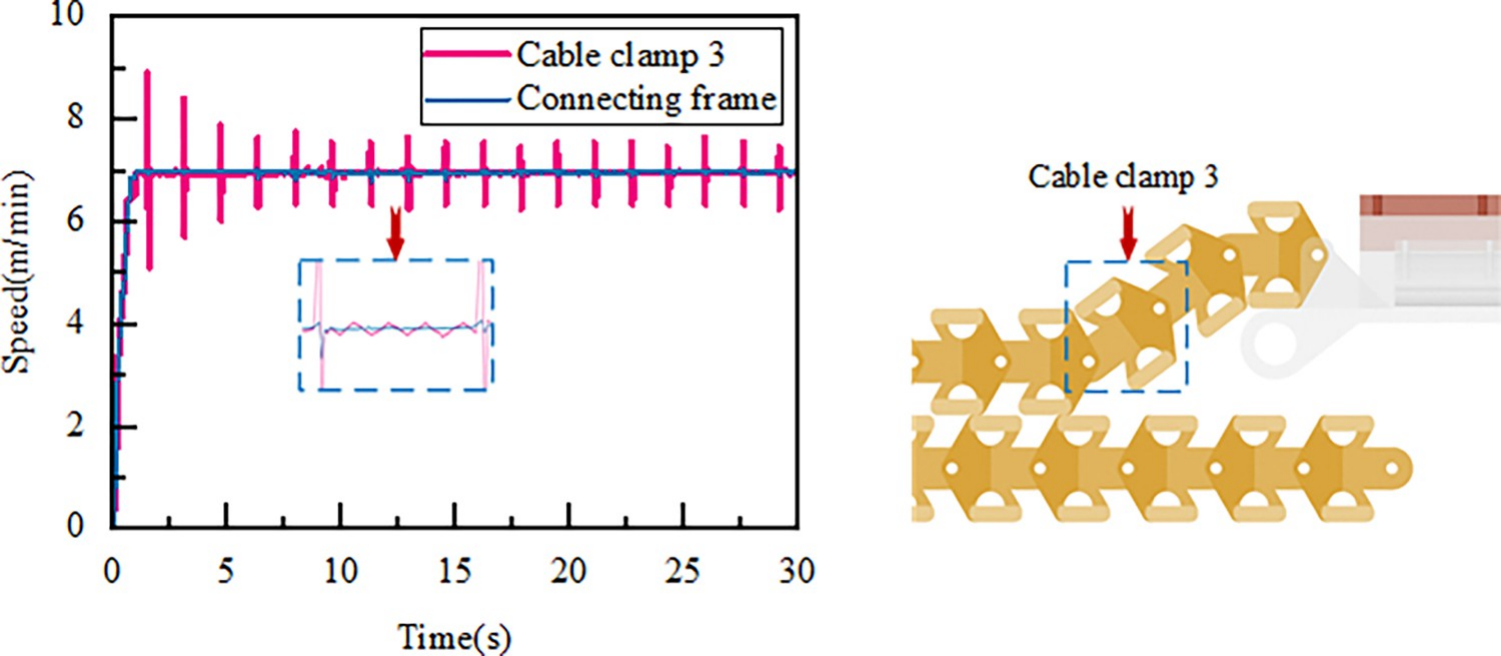
Upper layer cable clip 1, connection frame speed-time curve.

In the drag cable system, the cable clamps are divided into upper and lower layers. The motion characteristics of the upper-layer end cable clamp 3 and the connection frame are shown in Fig 6. The overall trend of cable clamp 3 is largely consistent with that of the connection frame, with secondary fluctuations appearing around the connection frame’s curve. Due to the partial suspension of cable clamp 3, its velocity exhibits the most significant fluctuations, peaking at 8.9 m/min at 2.2 seconds. As the system continues to operate, the magnitude of these fluctuations gradually decreases, stabilizing after 6 seconds, with the velocity fluctuating between 6.0 and 7.4 m/min.

**Fig 7:**
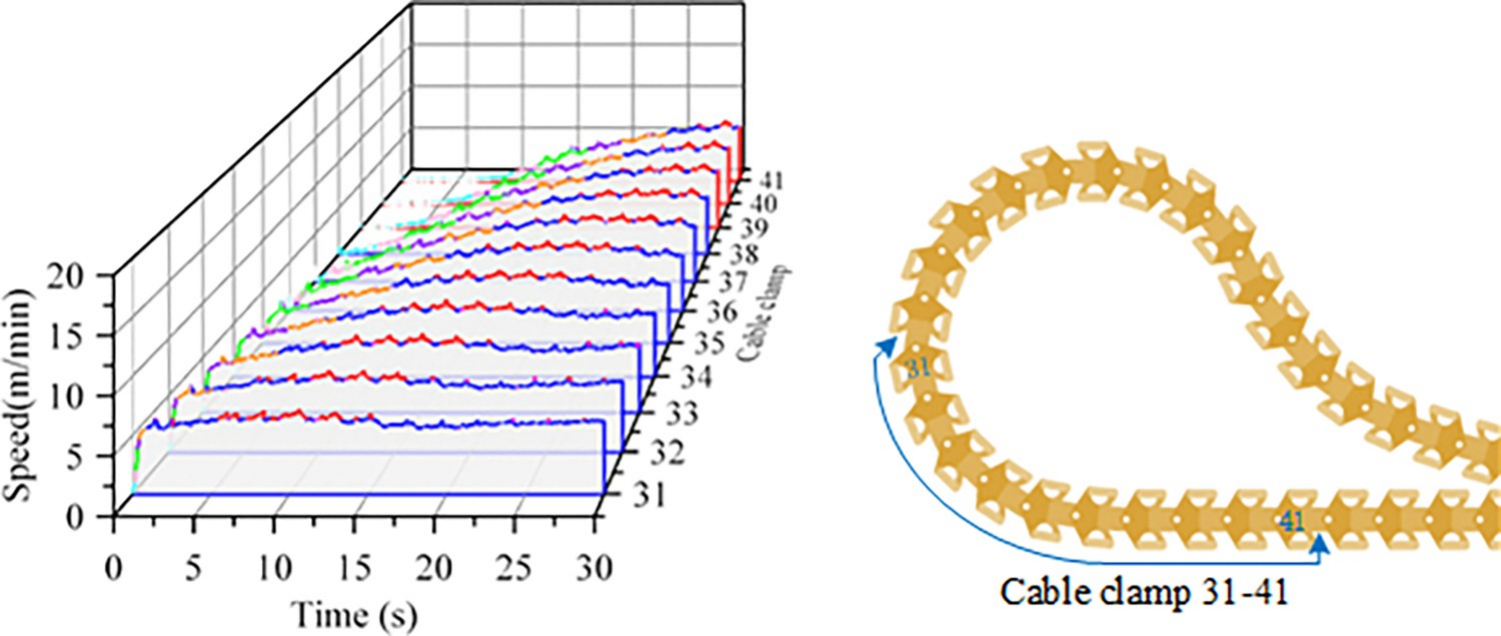
Cable clip 31–41 bending process speed-time curve.

The lower cable clamps enter a bending phase as the system operates. Ten consecutive clamps at the bending section were selected for analysis. The speed variation during the bending process is shown in Fig 7. As each clamp successively enters the bend, the drag speed gradually increases. Collisions between the clamps cause fluctuations in speed, with the maximum speed observed at the top of the roller, reaching 7.1 m/min. After exiting the bend, the speed stabilizes and fluctuates around 6.6 m/min.

**Fig 8:**
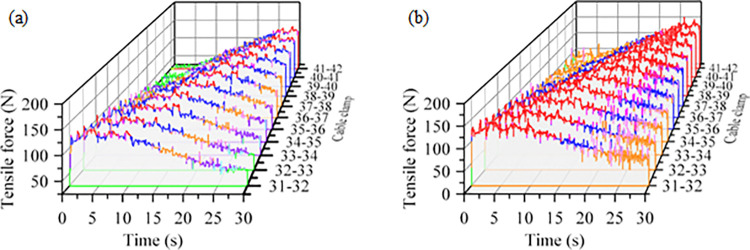
Cable bending tension between cable clips-time curve: (a) Dragging standard cables; (b) Dragging reinforced cables.

At the bending section, the cable clamps are influenced by both the internal cable and the external roller, resulting in more complex force conditions. Additionally, different cable types have varying effects, as shown in Fig 8. Table 6 presents the changes in tensile force between the clamps when dragging standard and reinforced cables through the bend. The average tensile force increases by 12.2 N when dragging reinforced cables compared to standard ones, and by 9.7 N after exiting the bend. Although the frequency of force fluctuations between the clamps increases during the dragging of reinforced cables, the overall trend remains similar to that observed with standard cables. By increasing the thickness of the flat plate on the upper and lower surfaces of the cable clamp and the curvature of the contact surface, the strength of the cable clamp and the contact area with the cable can be increased to adapt to the reinforced cable. By adjusting the cable core stranding pitch to reduce the force required for bending, can reduce the contact force with the cable clamp, in order to ensure the tensile strength at the same time, reduce the damage to the cable clamps [27].

**Table 6:** Table of tension between cable clamp when ordinary and reinforced cables bend.

Type of cable being towed	Before entering the bend	During bending	After exit bending
Standard cable	Fluctuates around 28 N	Increases with fluctuations to a maximum value of 133 N	Oscillating around 57 N
Reinforced cable	Fluctuates around 37 N	Increases with fluctuations to a maximum value of 146 N.	Oscillating around 66 N

As the length of the dragging cable increases, the tension experienced by the cable clamp at the connection point with the shearer also rises. The force exerted on the cable clamp is primarily concentrated on the steel framework. The curves were fitted after processing the high-frequency noise by FFT filter. In Origin, a cutoff frequency slightly higher than the primary frequency components of the signal is selected to retain the main features of the data. A window size of 12 points and a cutoff frequency of 1.65 Hz are set to remove high-frequency noise and analyze the fluctuation characteristics of the data. The data is then fitted to evaluate the overall trend in the cable clamp tension.

**Fig 9:**
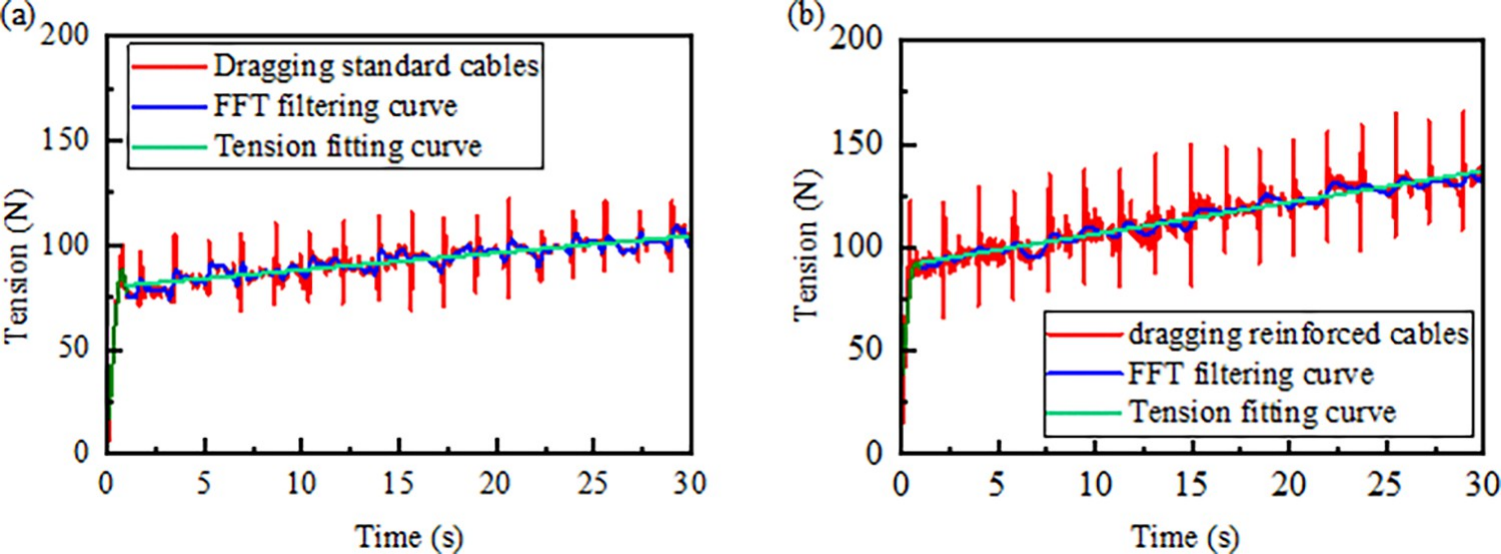
End cable clamping pull-time curve: (a) Dragging standard cables; (b) Dragging reinforced cables.

The tension trends for both ordinary and reinforced cables are similar, as shown in Fig 9 During the initial phase, as the system starts, the tension in the cable clamp gradually increases. Between 0.5 and 1.5 seconds, the upper reserved cable is dragged by the end cable clamp, resulting in an initial tension at the upper end cable clamp. During the system’s operational phase, the tension in the cable clamp steadily rises, with collisions between clamps causing fluctuations in the tension. In the later stages of the simulation, the tension continues to fluctuate upwards. Changes in cable clamp tension at the end of the towed ordinary and reinforced cables are shown in Table 7, with the maximum change in tension of the towed reinforced cable increasing by 37.3 N compared with that of the towed ordinary cable, and the average value of the overall tension increasing by 29.6 N. The tension and contact force fluctuations of reinforced cables are more pronounced. It is necessary to establish comprehensive selection guidelines for cable clamps and select appropriate clamps based on cable specifications. Additionally, optimizing the layout and spacing of the clamps ensures an even load distribution across the entire dragging system, thereby reducing fluctuations in tension and contact force.

**Table 7:** Drag normal and reinforced cable end clamp tension change table.

Type	System start-up phase	System operation phase	Post-operational phase of the system
Standard cables	Gradually increasing and fluctuating around 75 N	The fluctuations increased, with a maximum amplitude of 57.4 N	Fluctuations increased, oscillating around 106 N between 29s and 30s
Reinforced cables	Gradually increasing to fluctuate around 92 N	Fluctuations increased, with a maximum amplitude of 94.1 N	Fluctuations increased, oscillating around 127 N between 29s and 30s

Based on the actual working conditions of 5615 working face, the maximum values of pulling force were 925.2 N and 1134.7 N when the cable clamps dragged the ordinary and reinforced cables when the coal miner cut to the end of the working face, The value of the pulling force is much smaller than the pull-off force 70KN of the cable clamp [20], and the simulation results are in line with the actual working conditions of the cable clamp system operation [28]. The complex environment of the coal mining face makes variable-amplitude loads more prevalent in areas with severe fluctuations. The sustained tension fluctuations experienced by cables and cable clamps subject them to increased stress cycles, directly impacting their fatigue life and performance stability. When exposed to impact loads, the risk of damage or even breakage to the cables and clamps rises significantly, potentially causing power supply interruptions to the shearer and jeopardizing the safety and productivity of the coal mining operation.

### 4.2 Dynamic characteristics analysis of shearer cables

**Fig 10:**
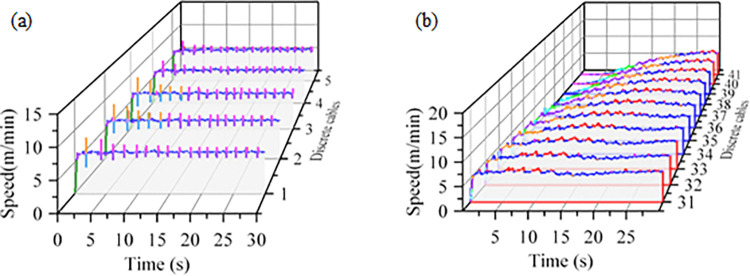
Discrete cable speeds at system ends and bends: (a) End Discrete Cables; (b) Discrete cables at bends.

The shearer cables are dragged by cable clamps, and the running speed of the upper end of the continuous discrete cables is illustrated in Fig 10A). Cable clamps 1 to 3 do not make contact with the lower cable clamps, resulting in a suspended area; thus, the velocity variations of the internal discrete cables are more pronounced. The end cable 1 is tightly pressed by the connecting frame’s pressing plate, limiting its range of motion, and its fluctuation amplitude is generally smaller than that of discrete cables 2 and 3. For discrete cables 3 to 5, the amplitude of fluctuations increases as they approach the connecting frame. At 1.6 seconds after system startup, the velocity of discrete cable 3 experiences the most significant fluctuations, peaking at 8.7 m/min. As the system continues to operate, the amount of velocity fluctuation gradually decreases, stabilizing between 6.0 and 7.2 m/min after 10 seconds.

During the cable bending process, the running speed changes as the cable position varies, as illustrated in Fig 10(B). When the discrete cables enter the bend, the velocity fluctuations increase, with a maximum variation of 1.3 N.

**Fig 11:**
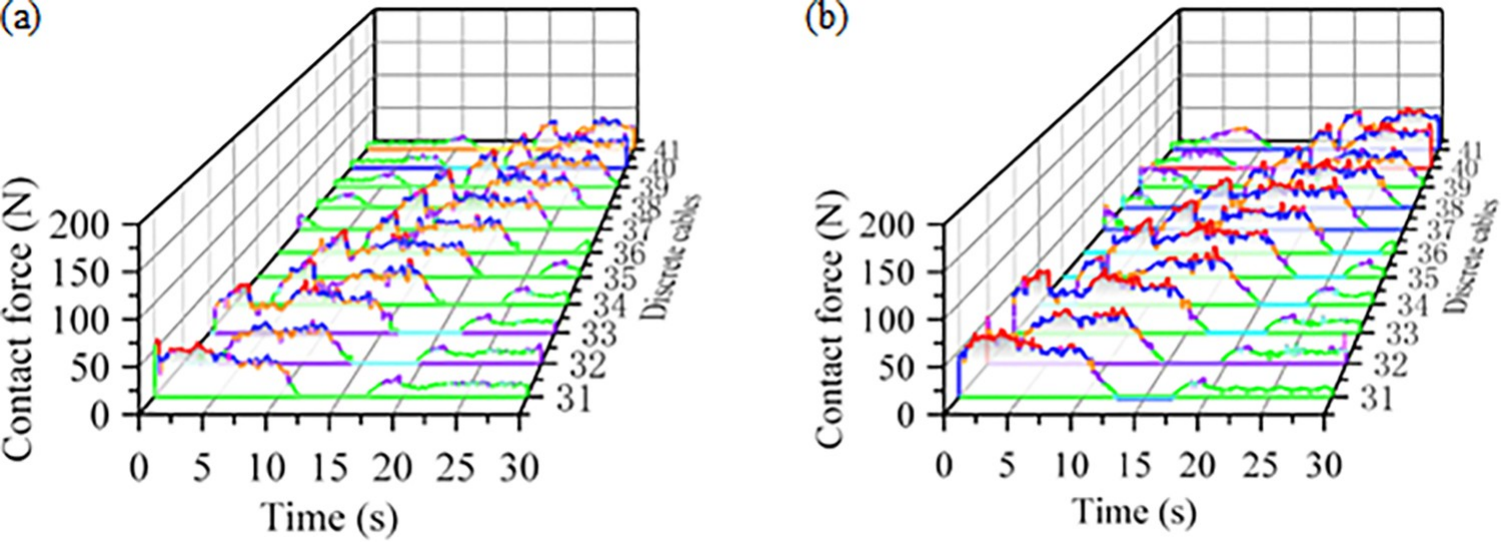
Bending process cable and cable clamp contact force-time curve: (a)Standard cable; (b)Reinforced cable.

The contact between the cable and the cable clamp causes bending, making the contact force crucial to the cable bending process. The variation in contact force between the cable and the cable clamp during bending is illustrated in Fig 11. The magnitude of the contact force is related to the bending angle, as shown in Table 8. During the bending process, the average contact force between the reinforced cable and the cable clamp increases by 22.3 N compared to the standard cable. This increase is attributed to the incorporation of steel wire inside the core and the metal braiding within the outer layer of insulation. As the cable exits the bend, the contact force gradually decreases to approximately 16 N and 17 N, allowing the cable to enter the upper layer for towing. The contact force between the cable and the cable clamp varies with the bending angle. An increase in contact force indicates greater pressure exerted by the clamp on the cable, leading to deformation and wear of the cable’s outer insulation. This effect is particularly pronounced when the bending angle approaches 90°, where the contact pressure becomes more concentrated, accelerating insulation wear and reducing the cable’s service life.

**Table 8:** Contact force between normal and reinforced cables and cable clamps during the bending process.

Type	Entering a bend	0°~90° bending process	90°~180° bending process	Exit the bend
Standard Cable	The force increases from 8.1 N to around 16.3 N, then decreases to 0 N.	The force gradually increases to a maximum value of 61.3 N, then abruptly drops to approximately 23 N near 90°	The contact force at the top of the roller gradually increases, fluctuating between 45.2 N and 51.7 N	The contact force fluctuates around 16 N.
Reinforced Cable	The force increases from 9.1 N to approximately 17.4 N, then decreases to 0 N	The force gradually increases to a maximum value of 86.2 N, then abruptly drops to approximately 25 N near 90°	The contact force at the top of the roller gradually increases, fluctuating between 66.3 N and 73.6 N	The contact force fluctuates around 17 N.

**Fig 12:**
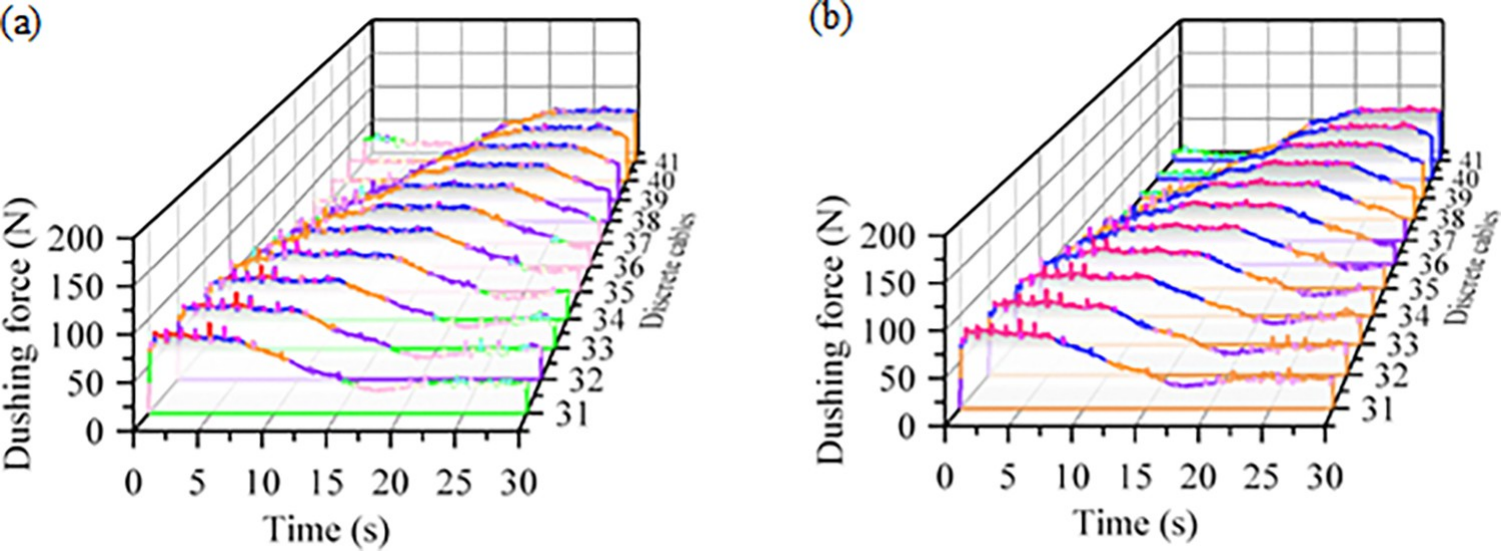
Bending process tension time curve between normal and reinforced cables (a) Tension between discrete standard cables (b) Tension between discrete reinforced cables.

The changes in contact force of the cable during the bending process are complex, necessitating a further analysis of the axial tension within the cable. The tension between the standard and reinforced cables during the bending process is illustrated in Fig 12. Prior to entering the bend, the tension between the discrete cables of the reinforced and standard cables is nearly identical. However, at the bend, the average tension in the reinforced cable increases by 18.7 N compared to that of the standard cable, as detailed in Table 9. The overall trend of tension fluctuations during the dragging of the reinforced cable is consistent with that of the standard cable. During the reciprocal bending of cables, the stress experienced by the strands of the power unit increases with proximity to the outer layer [27]. Cables subjected to fluctuating tensile forces over an extended period will undergo gradual weakening of their internal structure. Once internal damage reaches a critical level, the cable can no longer withstand normal operating stress, ultimately resulting in failure.

**Table 9:** Tension variation table between ordinary and reinforced discrete cables.

Type	Entering the Bending Process	Bending Process	Exit of the Bending Process
Standard cable	The force fluctuated, increasing from 6.2 N.	The force fluctuated around 58.4 N	The force decreased to fluctuate around 12.6 N
Reinforced cable	The force fluctuated, increasing from 9.3 N.	The force fluctuated around 80.5 N	The force decreased to fluctuate around 32.8 N

### 4.3 Impact of ring chain drive characteristics on the drag cable system

**Fig 13:**
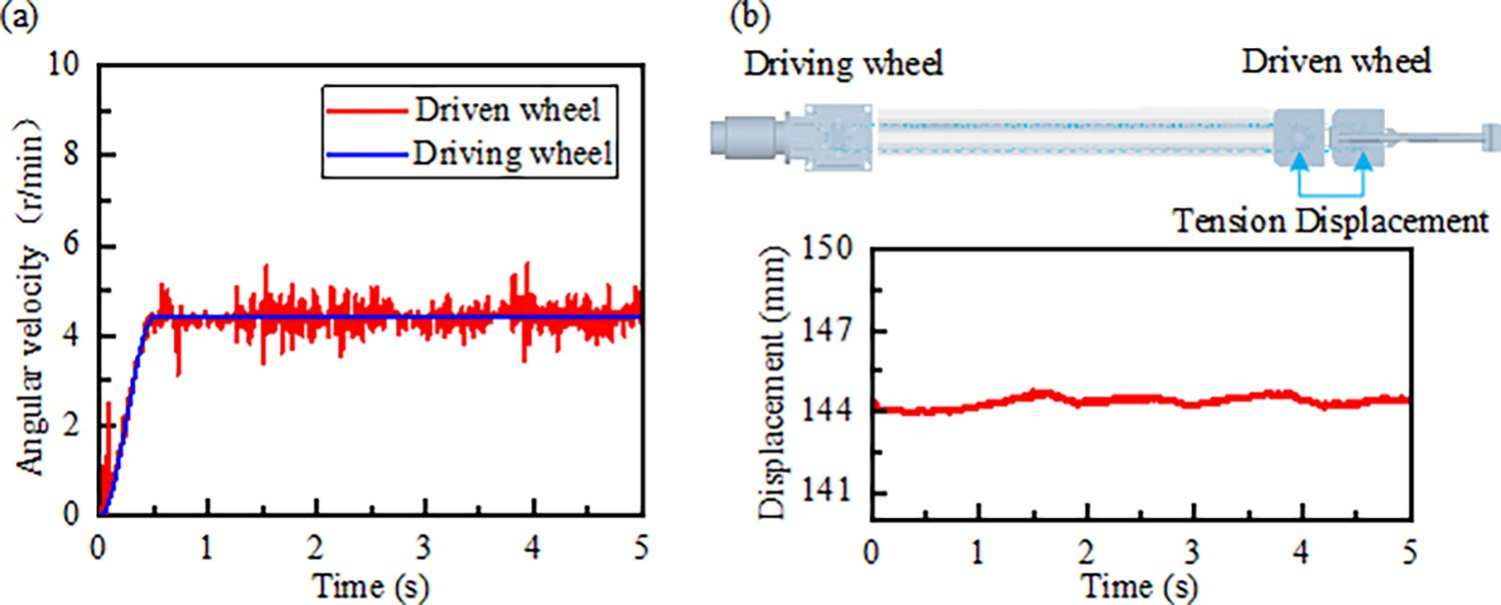
Kinematic characteristics of chain wheel: (a) Main and slave wheel angular velocity; (b)Driven wheel tension displacement.

The ring chain drive is the power component of the cable drag system, ensuring a single bending of the cable through the cable drag cart [29]. Upon system activation, the chain transitions from a slack to a taut state, resulting in fluctuations within the chain drive system [30]. The speed of the chain wheel is illustrated in Fig 13(A): the rotational speed curve of the driving wheel aligns with the drive function. Within the first 0 to 0.5 seconds, the speed of the driven wheel increases alongside the driving wheel, reaching 4.4 r/min. In the initial operation phase, the speed of the driven wheel exhibits fluctuations, peaking at 2.2 r/min at 0.1 seconds. Under the influence of the tensioning device, the amplitude of these fluctuations gradually decreases. From 0.5 to 5 seconds, the driven wheel speed fluctuates between 3.5 and 5.8 r/min, with the maximum speed occurring during the engagement process due to the polygonal effect and drag cable load affecting the chain drive system. The driven wheel operates stably under the tensioning device, and the displacement of the tensioning hydraulic cylinder’s piston rod fluctuates around 144 mm, as shown in Fig 13(B), providing a reference for the study of the chain drive tensioning hydraulic cylinder.

**Fig 14:**
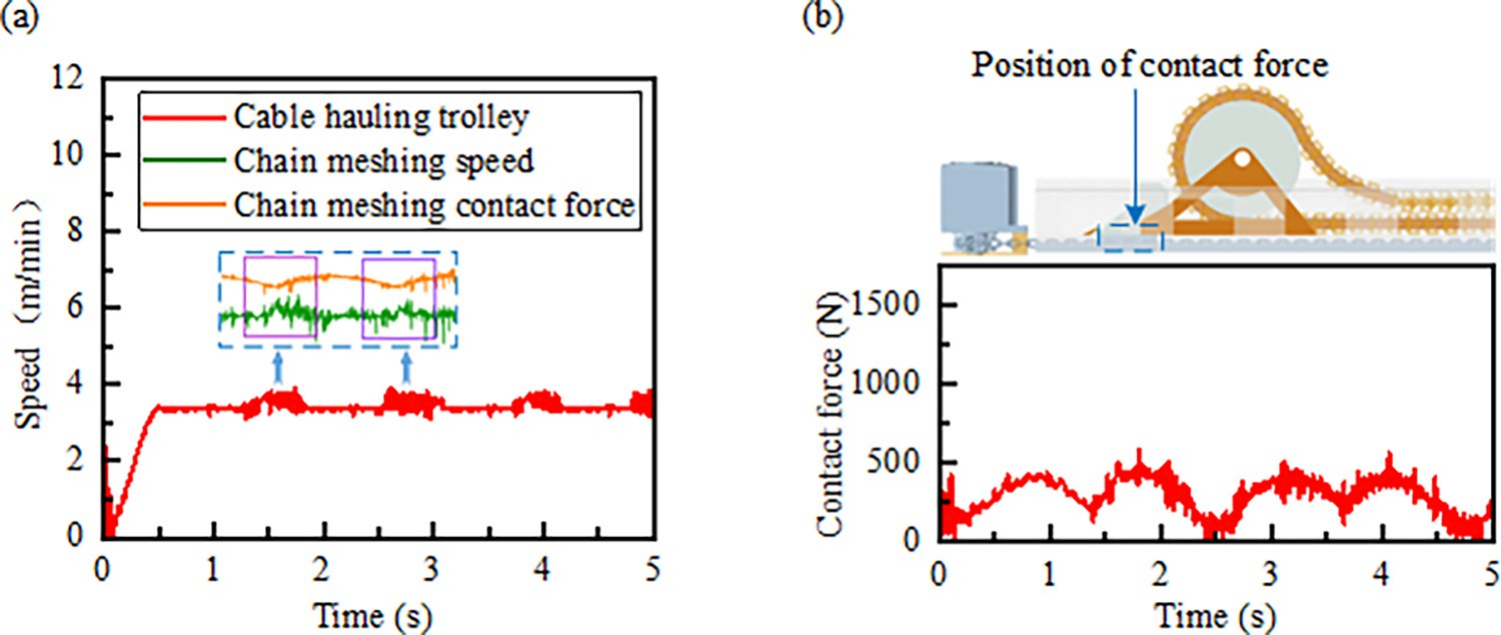
Kinematic characteristics of the cable drag cart: (a) Speed of the cable drag cart; (b) Contact force of the cable drag cart.

As the chain engages, the speed of the chain links and the contact force fluctuate periodically due to the meshing and disengaging of the links. This engagement affects the cable drag cart, causing periodic fluctuations in its speed, which remains around 3.3 m/min. Due to the mass of the cable drag cart, its speed fluctuations are smaller compared to those of the chain drive, as shown in Fig 14(A).

The cable drag cart is rigidly connected to the taut side of the chain, and it experiences periodic variations in contact force due to the polygonal effect of chain transmission. The maximum traction force reaches 562.9 N, as illustrated in Fig 14(B).

## 5. Conclusion

Based on the principles of equivalence and sleeve force theory, a mathematical model for solving the equivalent parameters of discrete cables was developed from the tensile test of the wire strands. The trajpar function was employed to construct an equivalent model of the power supply cable for the shearer, providing a reference for subsequent studies on the dynamic characteristics of shearer cables.

Based on virtual prototyping technology, a virtual prototype of the shearer cable drag system was constructed using macro commands. The simulation results indicate that, under the combined influence of drag resistance and the gap between cable clamps, the operational speed of the upper cable clamp fluctuates between 6.0 and 7.4 m/min. The tension between the clamps at the bend initially increases and then decreases, while the tension in the end clamp rises. The operational speed trend of the discrete cable is similar to that of the cable clamps. During the bending process, the contact force between the standard and reinforced cables and the cable clamps is related to the bending angle, reaching maximum values of 61.3 N and 86.2 N, respectively, as the angle approaches 90°. The tension between the discrete cables initially increases, stabilizes, and then decreases. Under the action of the tensioning device, the ring chain drive system can quickly recover stability from initial oscillations. The polygonal effect of the chain drive causes the traction force experienced by the cable drag cart to vary periodically, with a maximum value of 562.9 N. Based on the simulation results, future research should further optimize the design of the cable clamps to reduce the contact force at high bending angles, thus reducing the risk of cable damage. Meanwhile, complex signal pattern recognition of cable systems under high noise levels should be explored for fault diagnosis of coal mining machine cables. The construction of the virtual prototype model of the cable towing system provides a reference for intelligent cable towing research.

When dragging ordinary and reinforced cables, the differences in the system’s dynamic characteristics are primarily evident at the bending point and the connection between the system and the shearer. The inclusion of steel wires and metal braiding in the reinforced cables leads to an average contact force with the clamps that is 22.3 N greater than that of the ordinary cables. The average tension between the discrete cables is also 18.7 N higher for the reinforced cables. During the bending process of the reinforced cables compared to the ordinary cables, the average tension between the cable clamps increases by 12.2 N. At the connection point of the drag cable system and the shearer, the maximum variation in tension of the end cable clamps increases by 37.3 N when dragging reinforced cables compared to ordinary cables. When the shearer reaches the end of the working face, the maximum tensions are 925.2 N and 1134.7 N, respectively, which are well below the breaking force of the cable clamps. This research provides a reference for the optimization of shearer cable design.

## Supporting information

S1 Fig Tensile test samples of power core, ground core, control core: (a) Power and ground cores; (b) Control core. specimen 1, specimen 2, specimen 3, specimen 4, specimen 5. (XLSX)
